# Policy Preferences Regarding Health Data Sharing Among Patients With Cancer: Public Deliberations

**DOI:** 10.2196/39631

**Published:** 2023-01-31

**Authors:** Minakshi Raj, Kerry Ryan, Philip Sahr Amara, Paige Nong, Karen Calhoun, M Grace Trinidad, Daniel Thiel, Kayte Spector-Bagdady, Raymond De Vries, Sharon Kardia, Jodyn Platt

**Affiliations:** 1 Department of Kinesiology and Community Health University of Illinois Urbana Champaign Champaign, IL United States; 2 Center for Bioethics and Social Sciences in Medicine University of Michigan Ann Arbor, MI United States; 3 Department of Learning Health Sciences University of Michigan Ann Arbor, MI United States; 4 Department of Health Management and Policy University of Michigan School of Public Health Ann Arbor, MI United States; 5 Michigan Institute for Clinical & Health Research Ann Arbor, MI United States; 6 National Hemophilia Program Coordinating Center Ann Arbor, MI United States; 7 Lyman Briggs College Michigan State University East Lansing, MI United States; 8 School of Public Health University of Michigan Ann Arbor, MI United States

**Keywords:** public deliberation, data sharing, precision health, health information exchange

## Abstract

**Background:**

Precision health offers the promise of advancing clinical care in data-driven, evidence-based, and personalized ways. However, complex data sharing infrastructures, for-profit (commercial) and nonprofit partnerships, and systems for data governance have been created with little attention to the values, expectations, and preferences of patients about how they want to be engaged in the sharing and use of their health information. We solicited patient opinions about institutional policy options using public deliberation methods to address this gap.

**Objective:**

We aimed to understand the policy preferences of current and former patients with cancer regarding the sharing of health information collected in the contexts of health information exchange and commercial partnerships and to identify the values invoked and perceived risks and benefits of health data sharing considered by the participants when formulating their policy preferences.

**Methods:**

We conducted 2 public deliberations, including predeliberation and postdeliberation surveys, with patients who had a current or former cancer diagnosis (n=61). Following informational presentations, the participants engaged in facilitated small-group deliberations to discuss and rank policy preferences related to health information sharing, such as the use of a patient portal, email or SMS text messaging, signage in health care settings, opting out of commercial data sharing, payment, and preservation of the status quo. The participants ranked their policy preferences individually, as small groups by mutual agreement, and then again individually in the postdeliberation survey.

**Results:**

After deliberation, the patient portal was ranked as the most preferred policy choice. The participants ranked no change in status quo as the least preferred policy option by a wide margin. Throughout the study, the participants expressed concerns about transparency and awareness, convenience, and accessibility of information about health data sharing. Concerns about the status quo centered around a lack of transparency, awareness, and control. Specifically, the patients were not aware of how, when, or why their data were being used and wanted more transparency in these regards as well as greater control and autonomy around the use of their health data. The deliberations suggested that patient portals would be a good place to provide additional information about data sharing practices but that over time, notifications should be tailored to patient preferences.

**Conclusions:**

Our study suggests the need for increased disclosure of health information sharing practices. Describing health data sharing practices through patient portals or other mechanisms personalized to patient preferences would minimize the concerns expressed by patients about the extent of data sharing that occurs without their knowledge. Future research and policies should identify ways to increase patient control over health data sharing without reducing the societal benefits of data sharing.

## Introduction

### Background

Precision medicine is a growing effort to use state-of-the-art molecular markers and clinical decision supports to enable the customization of patient care. The first major successes have been in the field of precision oncology, where patient data (laboratory results, tumor pathology, treatment, survival time, etc) are routinely matched with the genome sequencing of tumors to enable cancer clinics, as well as pharmaceutical and commercial companies, to refine diagnostics and treatments to improve patient outcomes [1-3]. Although some precision oncology approaches have evolved under the regulatory standards associated with research, the vast majority of health data sharing and creation of new clinical regimes have occurred as part of the quality improvement processes, which are not subject to the regulations governing human participant research. Health data, which can be derived from biological, clinical, tracking, administrative, or patient registry information, are routinely collected from individual patients and shared electronically among doctors, nurses, hospitals, commercial laboratories and diagnostics, insurance companies, public health departments, and other information networks [4-6]. Sharing this information has become an essential component of care delivery and coordination as well as population health [7].

However, patients are generally unaware of the extent of data sharing that occurs in the context of health care delivery. Although the notification of data sharing policies is described in Health Insurance Portability and Accountability Act forms, health institutions fail to make them accessible to patients [8]. For instance, a study found that patients were not aware that the precision medicine biobank consent form they signed permitted the commercialization of their data; upon discovering this, both the patients and referring physicians expressed concerns about privacy [9]. This suggests that despite some of the benefits of health information sharing for advancing research and clinical care, the lack of transparency and privacy risks pose a threat to trust [10,11]. At a minimum, posting information in clinical settings in plain language would promote greater transparency in how health information is shared. Health organizations could also leverage the existing systems used to signal data breaches—via SMS text messages or a patient portal—to increase the awareness of data sharing practices; these systems could also be adopted by commercial companies. In addition to these strategies, patients could be offered the option to opt out of commercial data sharing entirely or be paid for the use of their data.

### Goal of This Study

We used a deliberative method to obtain a rich qualitative understanding of the key attributes of patient preferences for systems that share clinical health data in the context of precision oncology. Deliberation reveals the complexity and nuances that inform specific recommendations for the ethical governance of health information [12-14]. The objectives of this study were to apply the method of public deliberation to (1) describe the policy preferences of current and former patients with cancer regarding clinical health information sharing and (2) identify the values, as well as the perceived risks and benefits associated with health data sharing, that participants called upon when formulating their preferences.

## Methods

We conducted 2 public deliberation sessions with English-speaking adults who were either current or former patients with cancer in Southeastern Michigan in October and November 2019. The purpose of the deliberations was to learn about patient concerns and preferences about how health information should be used, shared, and regulated.

### Ethics Approval

This study was approved by the University of Michigan Institutional Review Board and was deemed exempt from federal regulations (ethics approval number: HUM00158768). All the participants provided written informed consent before participation. The participants were compensated with US $100 and were provided with breakfast and lunch.

### Participants

We recruited participants through a research platform and database developed and managed by one of the Clinical Translational Science Institutes designed to facilitate the recruitment of research participants [15]. The database contained a pool of approximately 48,000 individuals. The inclusion criteria for our study were as follows: the participants had to be comfortable with speaking in English, had to be aged ≥21 years, and had to have a former or current diagnosis of any type of cancer. We purposively recruited participants to ensure diversity in terms of race or ethnicity, age, education, and sex. Eligible participants who expressed interest were contacted by the recruitment coordinator. From previous deliberation studies, we found that approximately 75% of enrolled participants ultimately attend a public deliberation [16]. Of the 79 participants who were enrolled, 61 (77%) attended 1 of 2 deliberation sessions. Given factors such as space and logistical considerations, we conducted 2 deliberations, with approximately 30 participants in each. This allowed for effective large- and small-group discussions [17].

### Materials

We developed educational presentations and a booklet for the participants (Multimedia Appendix 1). The educational presentations provided an overview of how health information is collected, stored, and shared in general and with commercial companies and the ethical considerations associated with information sharing [17]. The booklet was mailed to the participants before the session and included a description of the study and key terms. These materials were developed iteratively by the study team, which included a community partner and liaison, who reviewed the materials for accessibility. We used a variety of approaches, including visuals, narrative text, and use case scenarios, to further support accessibility and understanding. The participants also completed predeliberation and postdeliberation surveys on health system use, identified versus deidentified health information, comfort with commercial and noncommercial health data sharing, and preferences about notification of health data sharing. The surveys were informed by our previous nationally representative surveys [4,10,18,19].

### Procedures

The purpose of a deliberative session is to convene members of the public to obtain their input about a particular topic (here, health information sharing), gain insight into how they understand the complexities surrounding the topic, and solicit their preferred options for policy [13,14,20]. There are many different ways of conducting a public deliberation; for instance, deliberations could be varying in duration (eg, 1 day vs 2 days) and may include components such as opinion polls and issue forums [21,22]. The current deliberation was guided by Kim et al’s [23] deliberative approach [16], and the procedures are further described in our previous publication [17].

**Figure 1:**
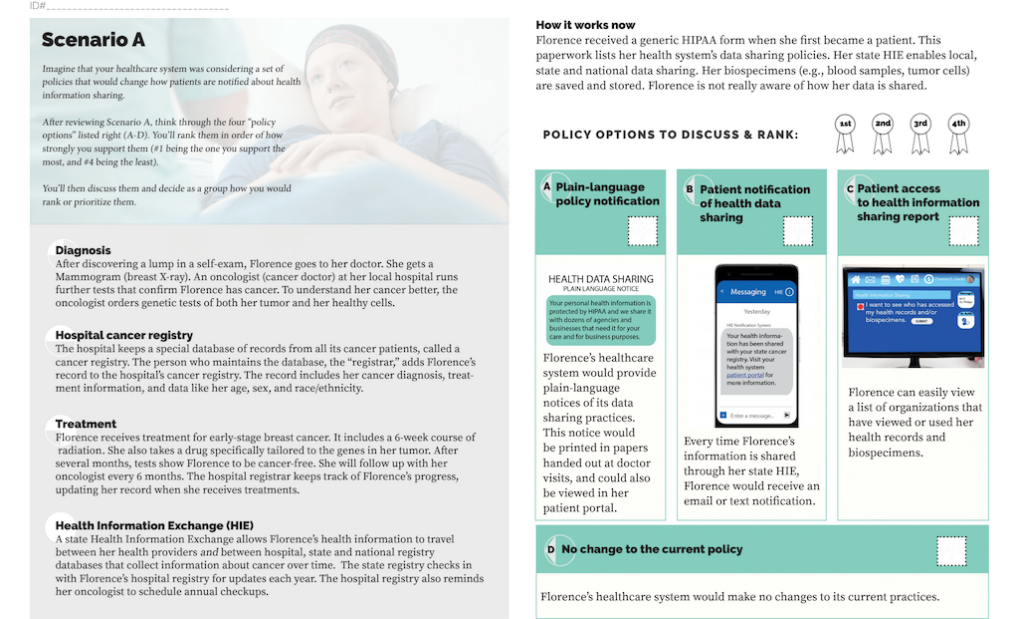
Scenario A policy options.

**Figure 2:**
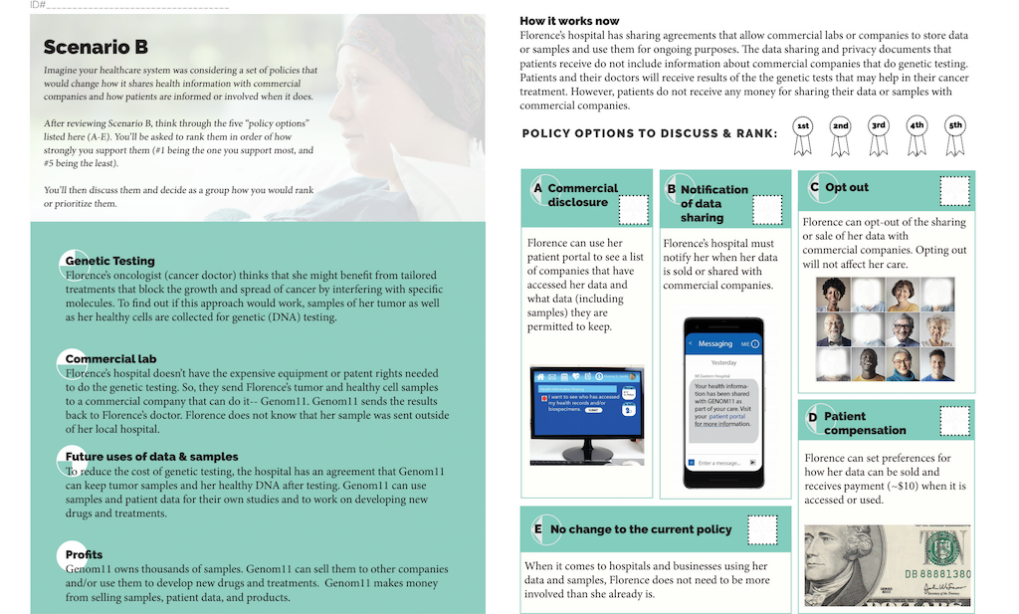
Scenario B policy options.

At the beginning of the day-long session, the participants completed a predeliberation survey, which included questions about knowledge and attitudes about data sharing as well as demographic information. The participants listened to presentations by experts on precision oncology, data practices, and the ethics of data sharing. They were randomly assigned to 1 of 5 small groups (6 to 8 people in each group) and participated in discussions led by trained facilitators. The goal of the small-group discussions was to have the participants rank a series of policy options related to 2 *scenarios* based on their preferences. In the first small-group session, scenario A, the participants were asked to deliberate over 4 policies for informing patients about clinical health information sharing. In the second small-group session, scenario B, the participants were asked to deliberate over 5 policies for informing patients about health information being shared with and used by commercial companies (Figures 1 and 2). These policy options were developed iteratively through discussions within the study team, which included members with expertise in policy, ethics, law, and precision oncology, and with health system and public health experts. The options were selected to balance feasibility and patient accessibility and were informed by previous literature considering different options such as payment for the use of health data [24]. Further information about our procedures and the deliberative process, along with the deliberation session agenda; the educational booklet given to the participants; and the postdeliberation survey can be found in our previous work [17].

At the beginning of each small-group discussion, the participants first ranked the options individually. These ranks were then reviewed and tallied in small groups to generate a score representing their group ranking. After a discussion focusing on the reasons for their preferences and the benefits and risks of each option for individuals and the larger society, the small groups had the option to revise their scores to come up with a final list of preferences. The group discussions also included, as needed, alternatives to or modifications of the policies as presented. This process was repeated for scenario B, which focused on the sharing of information with commercial companies. The participants then convened in a large group session to review and discuss the combined small-group results for both scenarios A and B. At the end of the session, the participants completed a postdeliberation survey, which included questions about knowledge and attitudes about data sharing as well as final individual policy rankings for scenarios A and B.

### Data Analysis

Our data analysis comprised a summarization of the participants’ demographic information and policy rankings and a qualitative analysis to assess the participants’ policy preferences and values and concerns related to health data sharing.

#### Participant Demographics and Policy Ranking

We collected demographic data from the presession survey and summarized them. Frequency, mean, and SD were calculated using SPSS (IBM Corp). We collected individual rankings before first small-group deliberation and in the postsession survey. The initial individual rankings informed the subsequent small-group discussions about policy preferences and concerns and benefits and risks of each option to individuals and the society. Small-group rankings were collected at the end of each small-group deliberation [17].

#### Qualitative Data Analysis

Audio recordings of the small-group discussions were professionally transcribed and deidentified. We used an iterative approach to design a codebook. An initial draft of the codebook was developed deductively based on previous deliberations on related topics [23,25,26] and our small-group discussion questions. Next, we had 4 members of the study team independently read through the 7 small-group discussion transcripts and suggest additional codes and edits to the existing codes. Three members of the study team tested and further refined the codebook via the double coding of 2 small-group discussions. The final codes reflected (1) policy preferences and (2) participant values and concerns related to health data sharing. Two members of the study team used the final version of the codebook to independently code all 20 small-group transcripts (10 from each session), after which they met to discuss and reconcile disagreements. The qualitative data analysis software MAXQDA 2018 (VERBI Software) was used for all analyses. The codebook is available in Multimedia Appendix 1.

## Results

### Participant Demographics

Table 1 summarizes the demographic characteristics of the participants. The mean age of the participants (n=61) was 62.1 (SD 10.2) years, and over half (36/61, 59%) of the participants identified as female. The reported race or ethnicity of the participants reflected the demographics of Southeastern Michigan residents: 72% (44/61) identified as White, 18% (11/61) identified as African American or Black, and 10% (6/61) as other races or ethnicities. Likewise, consistent with the community characteristics, just under half of the participants had a college (bachelor’s) degree (20/61, 33%) or higher level of education (25/61, 41%). Nearly three-quarters (45/61, 73%) were either working or retired. Over one-third (23/61, 38%) made less than the median household income of US $50,000. Most participants were in good health (42/60, 70% reported good or very good health status).

**Table 1:** Demographic characteristics of participants (n=61).

Characteristics			Values
**Sex, n (%)**			
	Female	36 (59)	
	Male	25 (41)	
Age (years), mean (SD)			62.1 (10.2)
**Race or ethnicity^a^, n (%)**			
	African American or Black	11 (18)	
	American Indian or Alaska Native	2 (3)	
	Asian American or Asian	2 (3)	
	Hispanic or Latino	3 (5)	
	Middle Eastern or Arab American	0 (0)	
	Pacific Islander or Hawaiian Native	0 (0)	
	White	44 (72)	
	Other	1 (2)	
**Highest educational qualification, n (%)**			
	Less than bachelor’s degree	16 (26)	
	Bachelor’s degree	20 (33)	
	More than bachelor’s degree	25 (41)	
Working in the health care field (yes), n (%)			16 (26)
**Household income (US $), n (%)**			
	<50,000	23 (38)	
	50,000-75,000	9 (15)	
	75,000-100,000	9 (15)	
	100,000-150,000	9 (15)	
	>150,000	5 (8)	
	Prefer not to answer	6 (10)	
**Employment status, n (%)**			
	Working	21 (34)	
	Not working (retired)	24 (39)	
	Not working (person with disability)	11 (18)	
	Not working (other)	4 (7)	
	Prefer not to answer	1 (2)	
**Health status^b^, n (%)**			
	Excellent	7 (12)	
	Very good	21 (35)	
	Good	21 (35)	
	Fair	10 (17)	
	Poor	1 (2)	

^a^The participants selected all the options that applied.

^b^Total number of participants is less than 61 owing to missing information from 1 (2%) participant.

### Ranked Policy Preferences

Across both scenarios, the participants ranked “No change to current policy” as the least preferred policy option. This was also reflected in the results of the individual postdeliberation survey, with the “No change to current policy” option obtaining a mean rank of 3.97 (SD 0.18) in scenario A (1 being the first rank and 4 being the fourth rank) and 4.46 (SD 0.92) in scenario B (1 being the first rank and 5 being the fifth rank). By contrast, the use of a patient portal was the most preferred policy option in both scenarios, although it was tied to the preference for email or text notifications in scenario B in the first deliberation session. Preference for the use of a patient portal was also reflected in the results of the individual postdeliberation survey, with the use of a patient portal option obtaining a mean rank of 1.46 (SD 0.59) in scenario A and 1.69 (SD 0.67) in scenario B.

In scenario A, other preferred notification options included the use of plain language signs and email or SMS text messages. The first deliberation session group preferred the use of email or text, whereas the second session group preferred the use of plain language signs. In the survey results, plain language was ranked slightly higher than email or text (2.18 vs 2.36) overall.

In scenario B, the participants also considered the option to opt out of sharing health information with commercial companies and the option to receive payment for the use of their data. In the first deliberation session, these 2 options tied. In the second session, opting out was ranked second, followed by notification via text or email and then payment. In the combined survey results, text or email ranked second overall (2.33), followed by opt out (2.85) and then payment (3.66). Table 2 summarizes the combined small-group policy preferences for each session and the mean ranks from the results of the individual postdeliberation survey.

**Table 2:** Small-group and individual survey rankings across both deliberation sessions.

Scenario and policy option		Rank in first deliberation session^a^ (n=28)	Rank in second deliberation session^a^ (n=33)	Mean rank (SD^b^; n=61)
**Scenario A: policy options for the sharing of clinical health information**				
	A.3. Disclosure: information posted on patient portal	1	1	1.46 (0.59)
	A.1. Notification: plain language signage	3	2	2.18 (0.85)
	A.2. Notification: text or email	2	3	2.36 (0.78)
	A.4. No change	4	4	3.97 (0.18)
**Scenario B: policy options for the sharing of clinical health information with commercial companies**				
	B.1. Disclosure: information posted on patient portal	1/2 (tie)	1	1.69 (0.67)
	B.2. Notification: text or email	1/2 (tie)	3	2.33 (0.94)
	B.3. Opt out of sharing with commercial companies	3/4 (tie)	2	2.85 (1.48)
	B.4. Payment	3/4 (tie)	4	3.66 (1.05)
	B.5. No change	5	5	4.46 (0.92)

^a^Final small-group ranking across the 5 small groups combined.

^b^On the basis on individual responses to the postdeliberation survey.

### Qualitative Findings

#### Overview

Across both scenarios, the participants felt that a change from the status quo is warranted, based on their hopes and concerns for individuals and the society. In their discussions, the participants weighed issues related to transparency and awareness, convenience, accessibility, individual autonomy and control, and respect. As the participants balanced the positives and negatives of each option, alternative solutions emerged, which we have described briefly in the subsequent sections.

#### Challenges With the Status Quo

There was little support for the status quo in either scenario A or B. The participants across both sessions agreed that the current policy is problematic because it lacks transparency, and subsequently, they said that they were unaware of their data were being shared:

I guess I just didn’t realize how much stuff was going out. That’s my biggest concern, and I’d like to be more aware of it.

Scenario A

In addition, in the context of commercial sharing (scenario B), the current policy does not allow for patient control over health data sharing:

Right now, we don’t know anything. We’re totally in the dark, and what we do know isn’t good. Basically, what we know now is if you want to be treated, then just sign all your rights away. It’s either that or don’t get treated, and that’s not really an option. Again, it’s not a choice.

Scenario B

No changes because Florence isn’t being given a choice about what her options are. She’s not even being informed of what her options are.

Scenario B

#### Hopes and Concerns: Individual and Societal Perspectives

##### Overview

Rationales for the need for a change from the status quo drew on participants’ concerns about the effects of the existing system on individuals and the larger society and hopes for a better approach. When considering both individual and societal levels, people were hopeful that information sharing would contribute to better cancer treatment and continuity of care; however, at the same time, they were concerned that sharing of data could lead to the denial of insurance coverage. Discussions also reflected individuals’ discomfort with not knowing about health information sharing (including how, with whom, and for what purpose it is shared) and with a general lack of control over how information is shared and used. The participants described several of such issues (eg, denial of insurance coverage and privacy and security), expressing concerns for themselves and others in the society. Other societal concerns were rising health care costs, discrimination and stigma and social injustice, public trust, and security. Simultaneously, the participants valued altruism and stressed on the importance of sharing information in the interest of benefiting all.

##### Individual Perspectives

The participants discussed personal reasons why they would support or have concerns about health data sharing in general. They perceived many benefits of sharing health data across networks, including the likelihood of personal benefit from cancer treatment because of previous health data sharing:

It may help me the next time, if I get [cancer] again.

Scenario A

They also saw value in improved communication among health systems facilitated by information sharing. For instance, one of the participants conveyed this as follows:

I guess the biggest benefit would be that it improves communication between healthcare systems...You don’t have to go to this doctor to get your MRI results and take them to this [other] doctor.

Scenario A

However, the participants were concerned about the personal risks associated with health data sharing, including potential discriminatory practices (eg, denial of health or life insurance) and the risk of private information being leaked to outside entities:

The main fear I have, and I don’t know if it’s real, but is an insurance company at some point getting my record and seeing I have a pre-existing condition and denying insurance to me. And I’d like to know other downsides besides that because when I think about the downside, that’s what always comes up for me.

Scenario A

The preference for options that notified patients about data sharing (email or text [Scenario A], portal [Scenarios A and B], and plain language signs [Scenario A]) was often stated by the participants in the context of a desire to know that their health information is being shared:

I would want the [email or text] notifications. It’s a high priority in terms of being advised if my information is distributed.

Scenario A

I chose disclosure [via patient portal] as my number one because I feel like I should have the option of knowing where my tissue is going and how it’s being used.

Scenario B

Simply because it’s [signs are] easy to read if it’s short and is in language that I understand and not in the medical terms...

Scenario A

However, there was also concern about a lack of detail in the plain language policy option, which involves putting signs in clinics and visible spaces about data use. For example, one of the participants conveyed this as follows:

While I like the idea of plain language and brevity, I also think it’s just too short. There’s just not...I mean it’s just... Boom. All of a sudden it’s like, “Well, you can get more information,” and I think it’s too plain and too easy and doesn’t really...In my opinion, it doesn’t protect my privacy and make me aware of my rights as a patient and as a consumer.

Scenario A

The participants described valuing control and rights over where their information goes, the nature of it, under what circumstances it is shared, and the implications of this sharing of data. They expressed emotions ranging from annoyance to anxiety and fear related to the life cycle of their data, which was seemingly out of their control. However, some were concerned that their preferences may ultimately not matter because their information was already “out there”:

In other words, the playing field as far as insurance, as far as healthcare is concerned doesn’t seem to be level at all. The people that’s making the rules don’t have to abide by the rules. So consequently, we’re caught between a rock and a hard place. So, whatever is going to happen to my medical records, it doesn’t do me any good to worry about it because it’s...already a done deal...

Scenario A

For others, the issue of identifiability was a meaningful concern, as they raised the question of how health information is shared (eg, deidentified vs identified) and what it would be used for (eg, research and care vs profit and commercialization). Some participants were concerned that deidentified data are not always truly deidentified. For example, one of the participants expressed the following*:*

Yeah, like if they found out there’s a 54-year-old guy in [hometown] with cancer on his neck...I mean, people would know it’s me.

Scenario A

However, other participants were comfortable with data sharing as long as the information was deidentified:

Yeah, I kind of see both sides. It’s like pharmaceutical. With some information, they can develop better treatments, better drugs, but at the same time they would have your...As long as things are de-identified, I don’t have a problem with sharing with whoever you want.

Scenario A

When discussing the potential for the commercialization of health information (scenario B), one of the most salient risks that the participants perceived was the lack of awareness of whether and with whom their information was being shared as well as the lack of control over its uses:

I think that it’s part of you or it was part of you, and you may not have any control over where it goes, but you should at least have the knowledge of where it’s going.

Scenario B

They were also troubled by the notion that commercial companies could be using their personal health information for profit with little obligation to the patient. Despite this, the participants were somewhat skeptical about the possibility of the system compensating them for their data through payment. Although our policy option proposed a US $10 payment, the participants wondered how to identify an appropriate valuation of their health data:

[I] for example, would want to use every opportunity to make that $10, but the next person would say, “I don’t need $10, even if it’s $100.”

Scenario B

Some participants worried that accepting payment for health data could be likened to “selling” themselves and presented a risk of compromising on privacy in the logistical aspect of actually receiving the payments; however, others felt that compensation to patients or data contributors might actually motivate companies to act more responsibly.

##### Societal Perspectives

Because of their personal experience with cancer, the participants were highly attuned to the role of the data life cycle in the development of treatments, advancement of research, and quality improvement. They described health data sharing as having the potential to help many other patients like them and were altruistic in their intentions, that is, they were willing to allow their health data to be used with nothing in return in hopes that it would be used to benefit others:

If that’s going to help somebody, then to me it’s somewhat worth it, regardless of who ends up with my records. But do I like it just being all willy nilly out there? No, I don’t, but I’m not going to lose sleep over the fact that it is. So, I think somewhere down the line, somebody is benefiting from it. Somebody is going to benefit from it, and that makes it somewhat more palatable for me.

Scenario A

My moral compass in all of that as “do something with it. Do something good. [Cancer] was a horrible thing. You got rid of it. So, make something good out of something bad.”

Scenario B

In fact, when discussing the possibility of the commercialization of health data, the participants valued the impact this approach could have on expanding treatment options to help patients like themselves. However, they did not trust that advancing research would be the extent of the data life cycle and thus saw many risks to society. One of the most common concerns was that insurance rates could increase because of greater access to information on individual health risks. They also described concerns about the risk of identity theft and discrimination, reflecting a broader societal perspective:

...it always used to be taxes and death were the two things you could...you know, you had to deal with and just couldn’t do anything about...so why bother fighting it. But it sounds like our health information is also now one of those things being free to everyone and anyone. To some extent, it’s a third now thing that you don’t have any control over. ...I guess what’s important is that there be teeth in people using it for reasons that end up being discriminatory. I’m more worried of that than anything else.

Scenario A

Beyond the issues of identifiability and privacy, the participants were concerned about the lack of transparency around procedures and the oversight and perceived lack of governance around the health data life cycle in general and for commercial purposes. These concerns were reflected in their policy preferences as well. For example, one of the participants described their concerns in this regard as follows:

I think [plain language] is not much better than [no change] because, once again, they’re telling you they’re sharing. You don’t know where it’s going. You have no control over where it goes. I think [notification] and [disclosure] give you the most knowledge about where your health information is being shared and gives you recourse if you don’t want it being shared with specific things...like [another participant’s] concern about insurance companies, and then once people are aware of where it’s going, then, yes, we can contact Congress and put pressure on them to enact laws that will give people more control over where their information goes.

Scenario A

However, the participants lacked the belief that commercial entities would be trustworthy in their use and sharing of health data, much less in reporting their uses of health data. The participants were especially struck by an expert presentation on the ethical implications of health data sharing and commented about their fear emerging from the historical misuse of health data, such as that experienced by the Havasupai Tribe in Arizona [27]:

I was frightened though when I saw that example of the Indian tribe that was...You know, their information was taken from them. The idea was sold to them that it would benefit A, and somebody used it for B, C, and D in a detrimental way. Maybe it’s helpful that they found the schizophrenic gene, but certain things do have a stigma to them. When you’re talking about a small group, an intimate group of a society, that could have a lot bigger effect than if they had said it to me.

Scenario A

How health information was used was of particular concern. Although some saw its use for advancing research and improving care as a primary benefit to the society, others were concerned about its potential misuse for profit:

I didn’t realize that tissue samples and vital information were being sold from company to company just for certain people to make money. The money should be rolled into real research that helps more people. ...I’d just like to see a system that had some dignity and respect for everybody in it, period, you know? We have certainly a checkered history of people being disrespected. If we could just learn from what has happened and try to remember...What’s shameful is to find out that even today dirty stuff is happening and people think they can cut corners and not notify and not respect and get away with it, and that hurts.

Scenario B

The potential for social injustices, ranging from discriminatory practices based on social identities and stigmatization of entire communities to the denial of individual health or life insurance, was salient in the participants’ conversations. Notably, this was not a consideration presented to the participants at the beginning of the session; they arrived at this concern on their own. They also wondered about the fairness of certain policies; for instance, they worried that older individuals with discomfort in using technology or individuals in areas without reliable internet access would not be able to engage in the email, text, or portal policy option. They also worried about the consequences and risks of injustices for others, including their biological relatives. For instance, one of the participants expressed her fear as follows:

One concern that I have is that, I had cancer at a young age, breast cancer, and so the implications for my girls is really...is really high, and I feel the ethical decision, “Do I find out more information for their sake,” or “do I protect them in a sense by not having the information and allowing them to choose when they want the information?”...I think that’s been my dilemma over the last...over the 15 years since I had cancer. Do I want to put the burden on them of knowing that they carry the gene? That is something that is going to be weighing on their shoulder every day. I know how it has affected one thing for me, getting life insurance. I can’t even get life insurance. Every time I try to get life insurance, it’s like, “How long have you had cancer? How long have you been cancer-free?”...You know, all this information that’s being shared is...It kind of scares me. It scares me for, their future.

Scenario A

The participants weighed these different values and individual and societal risks and benefits as they considered different policy options and compared their merits and challenges.

#### Specific Policies: Notification via Plain Language or Text or Disclosure via Patient Portal

##### Overview

In scenario A, the participants considered 3 different types of mechanisms for notifying patients about information sharing: posting signs in plain language in clinics and hospitals, sending SMS text or email messages to patients, and using a patient portal to notify patients about information sharing. In scenario B, which dealt with the sharing of information with commercial companies, email or text and patient portal options continued to be part of the deliberation. In both scenarios, 3 themes emerged as key considerations for the participants: the transparency and awareness of information sharing, convenience associated with each policy option, and accessibility of the policies.

##### Transparency and Awareness

In small-group discussions about their policy preferences, the participants primarily focused on the transparency issues in the system and their consequent lack or minimal awareness of data sharing practices when discussing the pros and cons of different policy options. The participants wanted policies to make patients aware of health data sharing, to make patients better understand health data sharing, and for organizations to be more open and provide details about health data sharing:

Okay, so [plain language] would have to be part of the package because this gobbily gook that we sign when we’re lying there in the emergency room ain’t no help, and it’s not telling you anything.

Scenario A

I chose the patient notification because I agree with having the information shared, and I just do want to know when it is shared with anyone else.

Scenario A

One of the reasons I chose the portal is because I could log into my chart and see all the information, and to me that’s very comparable to getting a free copy of my credit report every year.

Scenario A

##### Convenience

The participants were also asked whether the policy options were convenient or placed a burden on patients. They discussed issues regarding patient comfort, familiarity, ease of navigation, and simplicity and concerns about overloading patients leading to frustration and annoyance:

I guess it would be much easier just to get it on your phone versus a text message versus having to go into the file through the portal.

Scenario A

I guess it’s sort of like the portal answer on the other is to receive the information if they want it, but, you know, not get overloaded.

Scenario B

##### Accessibility

Finally, the participants considered accessibility issues. They discussed whether policies were inclusive and expressed concerns about individual- and community-level gaps in access:

I’m liking the plain language more and more because the generations above me have the least amount of access to the Internet, and they’re the ones that need the information the most.

Scenario A

I see it a text notification as an issue with the amount of senior citizens. ...the difficulty in people seeing the text messages or understanding it, and the inability to see the keyboard.

Scenario A

Everyone has access to the portal. There are public libraries where you can use the computer. Nobody is excluded from it, even if they don’t own a device.

Scenario A

#### Specific Policies: Opt Out of Data Sharing With Commercial Companies

The introduction of the opt out policy option had the participants more explicitly reflecting on individual and societal trade-offs, weighing individual control and ownership versus the societal benefits of research progress. When discussing the “opt out” option, the participants appreciated the autonomy granted by the policy option but expressed concerns about losing the opportunity to advance research and benefit society:

My gut tells me that everybody should have the right to opt out. My brain tells me that if we have that option, it is going to too much limit benefits to everybody.

Scenario B

I think there’s more of a personal benefit, you controlling your own stuff, but there’s more of a social risk, you know? The bigger society is affected. So it’s hard. You know, who’s more important? You to yourself or the greater good? Now that’s going to be totally...Everyone is going to have their own opinion on that one.

Scenario B

#### Specific Policies: Payment

The payment policy option, which ranked low overall, had the participants weighing the benefits of getting paid or profit sharing in a commercial context versus a host of concerns around commercialization, such as the lack of feasibility, loss of privacy, and “ick” factor of buying and selling health data. The participants had mixed views and concerns regarding payment:

I do like the payment to the patient because while that could reduce bad usage, probably not. It also sort of puts a price on you, and I don’t like...And that feels icky.

Scenario B

When patients seek compensation, they think, Well, it’s about time. All these other companies are making beaucoup bucks. Why can’t I? But, like I said, if you get on...You know, you’re sharing your information with 3,000 companies, you know, nobody can keep track of, you know, where your information is going and, you know, when you get payment for something.

Scenario B

#### Modifications

Across both scenarios, the participants suggested several modifications and suggestions to build on the policies presented. Among these, the main suggestions included the following: (1) combining the policies to increase accessibility and awareness; (2) greater emphasis on patient education on data sharing; and (3) greater control over data sharing, including the ability to opt in or out of specific types of data sharing:

Why couldn’t we do more than one option? ...I just think you cover your bases that way, of people who don’t have technology and people who do have technology. That way, it covers more of the society in general, and that way...Especially as far as notifications of portal or push, that could be an opt-in/opt-out to either way via the portal. You know, say I want a push or just put it on my portal type of thing.

Scenario A

To me, it’s all about education. Doing all of this, but it’s educating the public to know how and what they can access. So, again, that’s my focus is education.

Scenario B

## Discussion

In this study, we report findings from 2 public deliberation sessions conducted with patients with cancer to learn about their policy preferences related to health information sharing in general and for commercial purposes. The participants weighed complex information and identified trade-offs between individual- and societal-level issues in the process of reaching a prioritized set of preferences for policies that could govern clinical health information sharing [17,20].

### Perceived Risks and Benefits of Health Information Sharing

The participants expressed a range of concerns and benefits associated with health information sharing for precision health, from individual- to family- to community-level issues. For example, they had concerns related to privacy and to employment and health insurance discrimination. Our findings are consistent with concerns found in previous studies that suggest that the perceived risks of sharing health information extend beyond threats to privacy [28]. For example, when considering the potential for data sharing, including with commercial companies, the participants were frustrated that the commercialization of health data emphasized the lack of patient ownership and that companies could make money from something as personal and private as health information. These concerns about the commercialization of health data echo issues raised in multiple studies, wherein the participants expressed a willingness to share data for “public benefit” but lacked clarity about how commercial uses of data—likely for profit—could also benefit the public [29]. Moreover, they worried about potential repercussions, such as identity theft or collective harm to communities, given the far-reaching movement of health data beyond the context of their provider. Although most people may not be directly harmed because of a privacy breach, it is emblematic of the kinds of concerns voiced in the deliberations.

The patients also perceived benefits to health data sharing; in particular, they attributed their cancer treatment to the exchange of information about previous patients with cancer. They recognized that sharing their own information has the potential to benefit others, and the discussions reflected high levels of altruism, wherein many participants expressed a willingness to sacrifice the privacy of their health information if it meant that others could benefit in the future [30]. These findings are consistent with previous studies demonstrating the perceived benefits of health data sharing, including supporting knowledge about diseases, advancing science, and helping patients learn more about their health conditions [31]. Even in the context of commercialization, the participants acknowledged that scaling treatments could be beneficial to other patients.

### Policy Preferences and Implications

The participants agreed that the status quo of tacit notification about the extent to which health data are shared beyond the immediate context of their clinical encounters is insufficient and expressed, nearly unanimously, a preference that the health care system modify this practice. The desire for greater transparency and information about health data sharing was grounded in their personal and societal concerns and expectations for the health care system and is consistent with previous studies finding that individuals value transparency about the data sharing process and subsequent uses of data [32,33]. Discussions of the status quo made it clear that the patients felt that the current practices do not honor the core bioethical foundations of patient autonomy or respect for persons.

Alternative forms of notification, including via an SMS text or email message or disclosure via a patient portal, were the most supported policy options. Disclosure through the portal was agreed upon as the most preferred policy for informing patients about health information sharing because it has the potential to enable patients to see where their health information has gone and for what purpose it is being used, in a place and time of their choice. However, the participants noted limitations of this type of policy related to accessibility, with the identified barriers similar to those to notification via SMS text messages. In particular, the use of the patient portal requires patient comfort with technology and internet access, and it requires being notified that there is new information in the portal. SMS text messages may be slightly more accessible but may not go far enough; in other words, the participants expressed a preference for not only being notified but also being able to actually view information about the health data sharing process. These digital disparities have been discussed in previous literature; here, we found that digital disparities not only might prevent access to care but, when compounded with disparities in health literacy, also present a barrier to understanding where one’s health information is going and for what purpose it is being used [34-36]. Although notification and disclosure may be promising policy approaches, more work is required to understand the impact of such approaches on disparities and to identify alternatives to ensure equitable access to information on health data sharing practices. Indeed, such policies could be highly effective in increasing transparency, yet they may not fully address some of the issues raised related to commercialization. Notably, our participants were concerned about the equity implications of any policy aimed at increasing transparency about health information sharing.

Support for a proposed scheme to pay patients for the broad use of their data was mixed, and we identified several key nuances around payment that make it potentially complicated. For instance, a previous study found that the median consumer is willing to pay US $5 per month to maintain data privacy but expects US $80 to allow access to personal health information [24]. Here, we focused on the latter option of payment to allow access to health information. Some participants in our study viewed payment as a positive way to affirm the value of the contributions made by patients, whereas others were put off by the prospect of being “bought off.” Robust discussions around the appropriate monetary value of a single instance of data use (is US $10 sufficient?) were inconclusive but illustrated the challenge of devising a policy that would be accepted as fair without being coercive. In addition, some participants raised the concern that offering compensation would only create more confusion around who owns individual patient information and to what extent patients might retain any rights after such a transaction. The story of Henrietta Lacks was invoked by a number of participants in these exchanges around compensation, and some participants suggested that instead of paying individuals, companies that benefit from information sharing in a commercial manner could be encouraged or required to support patients at a collective level (eg, through donations to patient support networks or patient advocacy organizations) [37]. This would respond to the ways in which the sharing and use of health data may harm (or benefit) individuals but often impact groups or communities and the society as a whole. Furthermore, certain groups may be unfairly or inequitably solicited for their data or may feel coerced into sharing data for income at the risk of later harm [38]. Instead, considering data as a collective resource could inform the development of policies that govern data use in a way that ensures collective benefits and harm reduction [39]. Findings from our study are consistent with other studies that have shown patients’ concerns about the privacy of their information [11,28], need for understanding the motives of commercial companies, and desire for policies and procedures that enhance transparency about the purposes, risks, and benefits of data sharing and use [40,41].

Given the strong reactions of our participants to the status quo and their general prior lack of awareness of the extent to which health information sharing pervades precision medicine, it may be tempting to counter the various concerns patients that have about health data sharing with a major information campaign focused on transparency, either at the point of care or through other means. However, as sociologist Gil Eyal [42] cautions, “a transparency blitz coming after a long period of being relatively opaque does not inspire trust. The provision of information as part of routine interactions, responding with openness when the trusting party wants to know more, does inspire trust.” On a broad level, our study suggests a need for the reorientation of practice in precision medicine toward increased disclosure and transparency around information sharing practices. Introducing the idea of health data sharing arrangements in a patient encounter, in the office waiting room, or more proactively through a patient portal or notification system could help minimize the uncanny experience of learning ex post facto how far patient data travel.

### Limitations

This study has some limitations. First, this analysis focuses on the perspectives and priorities of patients with cancer, which may not represent the needs or preferences of the general patient population or the perspectives of patients with other specific conditions. Although this narrow scope is important for understanding one of the most active types of precision medicine, future work will need to expand to broader patient populations to ensure that policies and regulations are responsive to their concerns as well. Second, although the diversity of the participants in this study was appropriate for the geographic setting of the study, future research should account for variations in diversity in other regions. Larger, population-based studies will be critical to testing our findings in a sample that represents greater diversity in background characteristics, health conditions, and experiences. In addition, findings from this study are within the context of the US health care system; concerns raised by the participants (eg, those related to implications for insurance) may be different in other health care systems. Research in different health systems may reveal different themes and policy preferences.

Findings from our study suggest that the current approach is not working; therefore, policies that inform patients of the accessibility and use of their health information must be developed. However, the policies discussed in this study are only a first step. Beyond awareness and notification are questions of ethical data use and governance. Future policies will need to be explicit about the conditions under which and by whom health information can be used.

### Conclusions

The expansion of precision medicine challenges our current frameworks for ensuring patient autonomy and respect. Creating regulations and policies that respond to public preferences is critical to ensuring that precision health initiatives honor these core bioethical principles. Transparency through patient access to information about data sharing and notification may facilitate patient engagement, whereas commercialization without patient notification may threaten the trust in health care systems. Patients are concerned about personal benefits and risks as well as benefits and risks to the society in general and will likely support systems that can demonstrate a thoughtful balance between individual- and societal-level concerns. At the same time, ensuring the responsiveness of regulations for data sharing in precision medicine requires continued solicitation of patient perspectives, desires, and concerns.

## Appendix

Multimedia Appendix 1 Codebook.

## Data Availability

Data from this study are available upon request to the authors.
