# Supplementary material for: Policy Preferences Regarding Health Data Sharing Among Patients With Cancer: Public Deliberations
Source: JMIR Cancer. 2023 Jan 31;9:e39631. doi: 10.2196/39631 (PMC9929721; doi:10.2196/39631)
Supplement: Multimedia Appendix 1 [file cancer_v9i1e39631_app1.docx]

**Codebook**

**Trade-offs/Back and Forth**

Participant(s) mentions/discusses trade-offs between different values or policies. One or more participants have a back and forth (disagreeing or building on one another) about the values or policy tradeoffs. [Should be double coded with values and/or policies]

**Analogies/Examples**

Participant(s) use an analogy or a comparison between data sharing, a policy, or an aspect of data sharing with some other concept or experience for the purpose of explanation or clarification. Comparisons or analogies using participant’s own experiences or media examples.

**Scenario A: Plain language (A) Pro**

Participant presents reason(s) why Plain Language/Choice A/Choice #1 in Scenario A is preferred by self or society OR presents a “pro”/benefit/upside of policy (regardless of whether they support it or not). Not every mention of the policy just those supported by reasoning.

**Scenario A: Plain language (A) Con**

Participant presents reason(s) why Plain Language/Choice A/Choice #1 in Scenario A is not preferred by self or society OR presents a “con”/risk/downside of policy (regardless of whether they support it or not). Not every mention of the policy just those supported by reasoning.

**Scenario A: Notification/text (B) Pro**

Participant presents reason(s) why Notification/text/Choice B/Choice #2 in Scenario A is preferred by self or society OR presents a “pro”/benefit/upside of policy (regardless of whether they support it or not). Not every mention of the policy just those supported by reasoning.

**Scenario A: Notification/text (B) Con**

Participant presents reason(s) why Notification/text/Choice B/Choice #2 in Scenario A is not preferred by self or society OR presents a “con”/risk/downside of policy (regardless of whether they support it or not). Not every mention of the policy just those supported by reasoning.

**Scenario A: Pt portal (C) Pro**

Participant presents reason(s) why patient portal/Choice C/Choice #3 in Scenario A is preferred by self or society OR presents a “pro”/benefit/upside of policy (regardless of whether they support it or not). Not every mention of the policy just those supported by reasoning.

**Scenario A: Pt portal (C) Con**

Participant presents reason(s) why patient portal/Choice C/Choice #3 in Scenario A is not preferred by self or society OR presents a “con”/risk/downside of policy (regardless of whether they support it or not). Not every mention of the policy just those supported by reasoning.

**Scenario A: No change (D) Pro**

Participant presents reason(s) why No change/Choice D/Choice #4 in Scenario A is preferred by self or society OR presents a “pro”/benefit/upside of policy (regardless of whether they support it or not). Not every mention of the policy just those supported by reasoning.

**Scenario A: No change (D) Con**

Participant presents reason(s) why No change/Choice D/Choice #4 in Scenario A is not preferred by self or society OR presents a “con”/risk/downside of policy (regardless of whether they support it or not). Not every mention of the policy just those supported by reasoning.

**Scenario A modification/suggestion/new**

Suggestions for modifying, improving, changing or combining one or more (or wanting “all of the above”) Scenario A policy options or processes. Also, suggestions for a new or different policy in Scenario A.

**Scenario B: Disclosure/portal (A) Pro**

Participant presents reason(s) why Disclosure/portal/Choice A/Choice #1 in Scenario B is preferred by self or society OR presents a “pro”/benefit/upside of policy (regardless of whether they support it or not). Not every mention of the policy just those supported by reasoning.

**Scenario B: Disclosure/portal (A) Con**

Participant presents reason(s) why Disclosure/portal/Choice A/Choice #1 in Scenario B is not preferred by self or society OR presents a “con”/risk/downside of policy (regardless of whether they support it or not). Not every mention of the policy just those supported by reasoning.

**Scenario B: Notification/text (B) Pro**

Participant presents reason(s) why Notification/text/Choice B/Choice #2 in Scenario B is preferred by self or society OR presents a “pro”/benefit/upside of policy (regardless of whether they support it or not). Not every mention of the policy just those supported by reasoning.

**Scenario B: Notification/text (B) Con**

Participant presents reason(s) why Notification/text/Choice B/Choice #2 in Scenario B is not preferred by self or society OR presents a “con”/risk/downside of policy (regardless of whether they support it or not). Not every mention of the policy just those supported by reasoning.

**Scenario B: Opt out (C) Pro**

Participant presents reason(s) why Opt out/Choice C/Choice #3 in Scenario B is preferred by self or society OR presents a “pro”/benefit/upside of policy (regardless of whether they support it or not). Not every mention of the policy just those supported by reasoning.

**Scenario B: Opt out (C) Con**

Participant presents reason(s) why Opt out/Choice C in Scenario B/Choice #3 is not preferred by self or society OR presents a “con”/risk/downside of policy (regardless of whether they support it or not).

**Scenario B: Payment (D) Pro**

Participant presents reason(s) why Payment/Choice D/Choice #4 in Scenario B is preferred by self or society OR presents a “pro”/benefit/upside of policy (regardless of whether they support it or not). Not every mention of the policy just those supported by reasoning.

**Scenario B: Payment (D) Con**

Participant presents reason(s) why Payment/Choice D/Choice #4 in Scenario B is not preferred by self or society OR presents a “con”/risk/downside of policy (regardless of whether they support it or not). Not every mention of the policy just those supported by reasoning.

**Scenario B: No change (E) Pro**

Participant presents reason(s) why No change/Choice E/Choice #5 in Scenario B is preferred by self or society OR presents a “pro”/benefit/upside of policy (regardless of whether they support it or not). Not every mention of the policy just those supported by reasoning.

**Scenario B: No change (E) Con**

Participant presents reason(s) why No change/Choice E/Choice #5 in Scenario B is not preferred by self or society OR presents a “con”/risk/downside of policy (regardless of whether they support it or not). Not every mention of the policy just those supported by reasoning.

**Scenario B modification/suggestion/new**

Suggestions for modifying, improving, changing or combining one or more (or wanting “all of the above”) Scenario B policy options or processes. Also, suggestions for a new or different policy in Scenario B.

**Benefit/Risk to Individual**

Participant mentions/discusses:

· Risk/harm or benefit to individuals OR participant *minimizes* this risk/harm or benefit to individuals.

· Personal benefit or risk/harm

· Benefits or risks could relate to health, mental well-being, discrimination, stigma, etc.

*Code only if participant clearly sees something as a benefit or a risk/harm.*

**Benefit/Risk to Society**

Participant explicit mentions/discusses:

· Risk/harm or benefit to society, the larger community, patients in general, etc. OR the participant *minimizes* this risk or benefit to society/larger community

· Benefits or risks could relate to societal, public, or community-level health, well-being, discrimination, vulnerable populations, etc.

· Benefits or risks to others

*Code only if participant clearly sees something as a benefit or a risk/harm.*

**Commercial vs. non-commercial**

Participant mentions or discusses the distinction/differences (or lack of difference) between non-commercial vs. commercial organizations or commercialization of health data . Or participant explicitly includes/excludes commercial companies in discussion about health data sharing.

**Control/Rights**

Participant mentions/discusses:

· Ownership of or control of own access to health data

· Control or lack of control over the process of health data sharing

· Lay public, patients, providers, health care organizations, commercial companies, government, or others don’t/can’t/shouldn’t control **OR** do/can/should have control over health data sharing.

· Freedom/autonomy to make a choice about health data sharing,

· Lay public, patients, providers, health care organizations, commercial companies, government, or others have or do not have rights, interests, or authority **OR** should or should not have rights, interests, or authority over health data sharing

· Giving consent (or not) to health information sharing

· Patient empowerment or control

The information is already out there and beyond the control of patients and the lay public

**Emotional impacts**

Participant mentions/discusses (negative or positive) emotional impacts of policies or data sharing:

· Worry about potential negative impacts on cancer patients and other vulnerable populations

· Feelings of distress, anxiety, annoyance, or being overwhelmed

· Protecting self, family, and others from harm, discrimination, distress, or discomfort

· Excitement or enthusiasm about possible positive impacts health data sharing

Emotional impacts of the potential consequences of health data sharing (ex. stigma/discrimination or research advances/better health)

**Future/hope/uncertainty**

Participants mentions/discusses the future (concern, hope, uncertainty) as it relates to health data sharing or impacts of health data sharing or health data sharing policies.

**Identifiable vs. Deidentifiable**

Participant mentions or discusses the distinction/differences (or lack of difference) between identifiable and de-identifiable data. Usually also coded under privacy.

**Knowledge/Awareness**

Participant mentions/discusses:

· Lay public or patients are OR are not informed/aware of/ knowledgeable about health data sharing

· Lay public or patients do OR do not know about/understand health data sharing process

· Lay public or patients want OR do not want to be informed/know about health data sharing

· Organizations should/should not OR do/don’t make us aware or share knowledge or provide information about health data sharing.

· Lay public or patients want to be more informed/knowledgeable about health data sharing

· Information overload/confusion

· Literacy, or specifically, health literacy

**Practicality**

Participant mentions that an action, perspective, or policy option is (*or is not):*

· practical/feasible/convenient

· technologically or administratively doable

· Or, obstacles can or cannot be overcome.

· Cost-effectiveness

- Ease of access (when not re: discriminatory barriers to access)

**Privacy/security**

Participant mentions/discusses issues of privacy, data security, others knowing or not knowing your personal information, hacking, etc.

· Confidentiality

· Disclosure of information

· De-identification/identification

· HIPAA

**Profit/money**

Participant mentions/discusses:

· Profit/money making by institutions/companies

· Cost/expenses by companies/institutions/others

· Institutions/companies/others making money off of healthcare information

· Individuals should or should not be compensated

*Not direct pros/cons/reasoning related to Payment policy in Scenario B. Those should be coded in Scenario B codes.*

**Responsibility/Governance**

Participant mentions/discusses:

· Who is or who is *not* or *should* *not* be responsible to protect the interests of patients and the lay public (such as patients themselves, healthcare organizations, oversight committees, the government, etc.) regarding health data sharing

· Who is responsible for making sure patients and the lay public are aware of and understand health data sharing

· Stewardship, governance, regulations as it related to protecting patients and the lay public regarding health data sharing

· Laws about data sharing; Legal responsibilities or liabilities

· References to duty, obligation, accountability, or IRB

**Trust/Transparency**

Participant mentions/discusses:

· Trust/distrust/transparency of healthcare organizations, providers, commercial companies, oversight, or other organizations or individuals.

· Something will either increase/decrease trust/transparency or create trust/transparency/distrust.

· References to honesty, openness, confidence, having faith in, suspicion, wariness, misgivings, cynicism, or doubts

**Willingness/Comfort**

Participant mentions/discusses:

• Own or others’ willingness/comfort (or not) with sharing health data

• They or general public care OR don’t care/are OR are not interested/concerned what happens to their health data

• Indifference about data being shared
